# Channel Formation in Cry Toxins: An Alphafold-2 Perspective

**DOI:** 10.3390/ijms242316809

**Published:** 2023-11-27

**Authors:** Jaume Torres, Wahyu Surya, Panadda Boonserm

**Affiliations:** 1 School of Biological Sciences, Nanyang Technological University, 60 Nanyang Drive, Singapore 637551, Singapore; 2 Institute of Molecular Biosciences, Mahidol University, Salaya, Phuttamonthon, Nakhon Pathom 73170, Thailand; panadda.boo@mahidol.ac.th

**Keywords:** Cry toxins, Alphafold, pore formation

## Abstract

*Bacillus thuringiensis* (Bt) strains produce pore-forming toxins (PFTs) that attack insect pests. Information for pre-pore and pore structures of some of these Bt toxins is available. However, for the three-domain (I-III) crystal (Cry) toxins, the most used Bt toxins in pest control, this crucial information is still missing. In these Cry toxins, biochemical data have shown that 7-helix domain I is involved in insertion in membranes, oligomerization and formation of a channel lined mainly by helix α4, whereas helices α1 to α3 seem to have a dynamic role during insertion. In the case of Cry1Aa, toxic against *Manduca sexta* larvae, a tetrameric oligomer seems to precede membrane insertion. Given the experimental difficulty in the elucidation of the membrane insertion steps, we used Alphafold-2 (AF2) to shed light on possible oligomeric structural intermediates in the membrane insertion of this toxin. AF2 very accurately (<1 Å RMSD) predicted the crystal monomeric and trimeric structures of Cry1Aa and Cry4Ba. The prediction of a tetramer of Cry1Aa, but not Cry4Ba, produced an ‘extended model’ where domain I helices α3 and α2b form a continuous helix and where hydrophobic helices α1 and α2 cluster at the tip of the bundle. We hypothesize that this represents an intermediate that binds the membrane and precedes α4/α5 hairpin insertion, together with helices α6 and α7. Another Cry1Aa tetrameric model was predicted after deleting helices α1 to α3, where domain I produced a central cavity consistent with an ion channel, lined by polar and charged residues in helix α4. We propose that this second model corresponds to the ‘membrane-inserted’ structure. AF2 also predicted larger α4/α5 hairpin n-mers (14 ≤*n* ≤ 17) with high confidence, which formed even larger (~5 nm) pores. The plausibility of these models is discussed in the context of available experimental data and current paradigms.

## 1. Introduction

### 1.1. Cry Toxins

Cry toxins are produced by *Bacillus thuringiensis* (Bt) and attack diverse insect species and other invertebrates. These toxins are highly specific and environmentally friendly [1] and have been used to control lepidopteran insect pests in agriculture and against dipteran insects that are vectors of human diseases [1,2], e.g., Dengue, Zika, or Yellow Fever [3,4]. In general, almost 800 members in the Cry toxin family [5] can be produced as 7-domain protoxins of about 130 kDa [6] that form parasporal crystals. Ingestion by susceptible larvae leads to crystal solubilization inside the larval gut. Protoxins may then bind to specific receptors in the apical microvilli membrane of the midgut cells [7] or are cleaved by midgut proteases into ‘activated’ three-domain Cry toxins (~60 kDa). These toxins bind to receptors located at the midgut brush border membrane (BBM) surface, e.g., aminopeptidase (APN), alkaline phosphatase, cadherin (CAD) or ATP-binding cassette transporters [8,9,10,11], and form an oligomeric pore, leading to osmotic shock, cell burst and death of the larvae. However, another mechanism has been proposed that apparently does not require oligomer formation, where toxicity arises when the toxin monomer sequentially activates a cell signaling pathway, leading to cell cytoskeleton destabilization [12].

### 1.2. Structure of Cry Toxins

The high-resolution structure of several Cry toxins has been elucidated in their activated, three-domain form, e.g., Cry3A [13], Cry4Ba [14,15], Cry4Aa [16], Cry1Aa [17], Cry2Aa [18], Cry3Bb1 [19], Cry8Ea1 [20], Cry5B [21,22], Cr7Ca1 [23], Cry1Da [24] and Cry11 (Aa and Ba) [25]. In all cases, the N-terminal domain I is formed by seven α-helices, α1 to α7 [13], and is involved in oligomerization and pore formation [14,16], whereas domains II and III contain mostly β-strands and are important for binding to receptors and in structural integrity [26]. Since these structures were solved in the absence of membranes or receptors, the oligomeric behavior and subsequent conformational changes that take place after binding to receptors and membrane insertion are still shrouded in obscurity. The latter information is important to develop Cry toxins that are more effective and can overcome resistance, or that are active and specific against novel targets. 

### 1.3. Interaction of Cry Toxins with Membranes

A number of models have been proposed for the assembly of the Cry toxin complex and its insertion into the membrane (see [1,27] for a recent review). The first model to explain toxicity in Cry toxins was the ‘umbrella model’ [28], where only a hairpin formed by domain I helices α4 and α5 inserts into the membrane. This model implicitly assumes that (i) at some point, these two helices move away from the rest of the α-helical bundle, and (ii) an unknown number of these hairpins associate to form a pore or channel, while the rest of the protein remains at the membrane surface [28,29,30,31,32,33]. Consistent with this model, synthetic peptides corresponding to helices α4 and α5 were claimed to be the only ones that could be inserted into phospholipid membranes, and the kinetics of insertion for peptide α5 indicated aggregation within the membrane [34,35]. Further, mutagenesis and biophysical data suggest helix α4 as a prime candidate to line the lumen of this pore [31,32,33,34,35,36,37,38]. However, although several experiments are consistent with a conformational change involving the separation of helices α1−α3 from the rest of the bundle in domain I [39,40,41,42,43,44,45], the separation of helices α4 and α5 from helices α6 and α7 is incompatible with experiments showing that bonds between helix α5 and α6 (or with α7), or between helix α7 and domain II, do not affect toxicity [39]. 

### 1.4. Proposed Pre-Pore Oligomer

The assay to determine the ability of Cry toxins to form oligomers after interaction with membranes consists of (i) separation of the membrane fraction after exposure of the toxin to liposomes or native membranes and (ii) solubilization of this fraction with SDS, followed by electrophoresis. By using this approach, Cry proteins have been suggested to form either trimeric or tetrameric oligomers (200–250 kDa) after interaction with receptors [46], although larger sizes have been reported, e.g., in Cry1Ia [47]. The size of these oligomers has been correlated with various parameters, e.g., the toxin ‘form’ (protoxin or activated toxin), the binding to a receptor or a membrane, or the type of receptor [46,48,49]. It has also been suggested that the pre-pore oligomer formed from the protoxin is more heat-resistant [50]. However, the significance of these oligomeric forms is not entirely clear; even though the treatment is mild, the native form existing in membranes for the pre-pore or pore structures may be too weak to stand SDS treatment. In fact, oligomers have been observed also for samples in solution, i.e., in the absence of lipid membranes [46,51,52]. Additionally, some toxic mutants, which therefore should form oligomers in membranes, only produced monomers in this assay, e.g., Cry1Ac H168R [35]. Thus, it is possible in our opinion that the oligomers observed in SDS may not represent those found in membranes, or they may represent smaller fragments of a larger oligomer. Tetrameric oligomers have been observed after incubation of Cry toxin with a peptide that mimics the cadherin receptor [35,36]. These tetramers were found to be more active than the monomeric form, both in insertion and in pore formation [53,54]. Additionally, Cry1Aa has been shown to form oligomers in membranes up to the level of tetramer using single-molecule fluorescence studies, although trimers were also present even at high toxin concentrations [55]. Cry toxins can also form trimers after exposure to brush border membrane vesicles (BBMVs) [37,38,56] or synthetic-lipid liposomes [57], and trimers or tetramers have been observed using Atomic Force Microscopy (AFM), electron microscopy and 2D crystallography [46,51,52,58,59,60], but whether these forms represent pre-pore or pore conformations is unclear.

Herein, we used Alphafold 2 [61,62] (AF2) to shed light on the possible models of Cry toxin. We chose two example representatives, one lepidopteran-specific (Cry1Aa) and one dipteran-specific (Cry4Ba). Both have a high-resolution crystal structure available, are two of the most studied Cry toxins over the years [14,43,45,55,57,63,64] and show a high degree of structural similarity, particularly with regard to the pore-formation mechanism, crucial for both toxins to kill their target pests. 

## 2. Results

### 2.1. AF2 Prediction of Cry Toxin Structure

**Figure 1:**
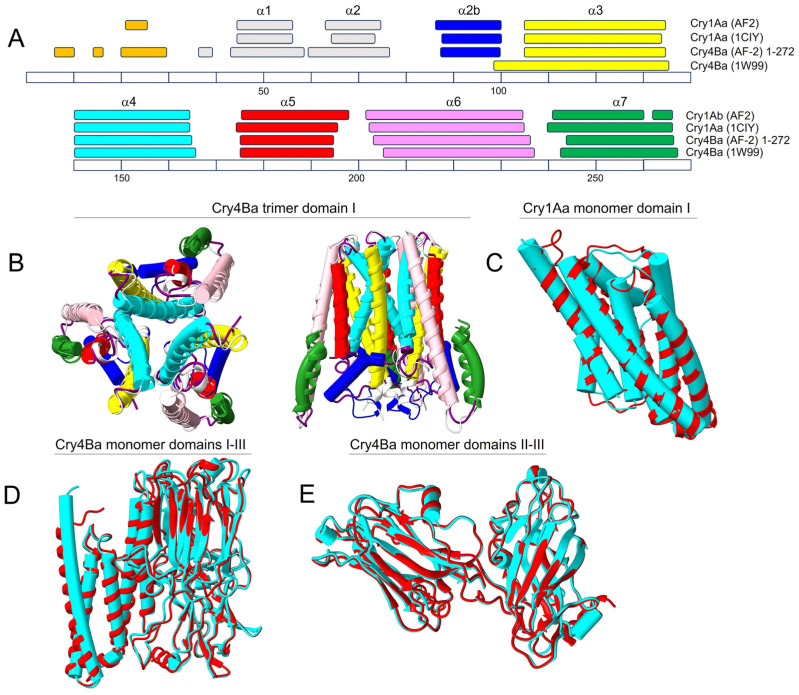
**Comparison between experimental and AF2-predicted structures:** (**A**) Comparison of experimental and AF2-predicted α-helical stretches (helices α1 to α7) in domain I for Cry1Aa and Cry4Ba. Each α-helical stretch has been color-coded in this and following figures for easy visualization. The sequence numbering shown at the bottom corresponds to the Cry4Ba sequence, whereas in Cry1Aa, the position of the α-helical segments were aligned with those of Cry4Ba, e.g., helix α3 spans residues 106–136 in Cry4Ba, but residues 90–120 in Cry1Aa; (**B**,**C**) overlay of experimental (cylinders) and AF2 structures (ribbon) of trimeric domain I in Cry4Ba (top and side views) (**B**) and for monomeric domain I of Cry1Aa (**C**). A trimeric model for domain I of Cry1Aa is shown in Figure 2A; (**D**) Cry4Ba monomer including domains I-III (cyan: experimental; red: AF2); (**E**) same as (**D**) without domain I, removed for clarity. The structures were fitted using Matchmaker in Chimera X [67].

We first tested whether AF2 could produce reliable structures of Cry toxins by comparison with published crystal structures, using Cry1Aa [17] and Cry4Ba [14] (crystallized as a monomer and as a trimer, respectively). Both sequences were tested as monomers and as homotrimers, using either domains I-III, or domain I alone. For both toxins, the prediction of domain I was identical, whether using domain I alone or domains I-III. For domain I of Cry1Aa, the length and position of the α-helical stretches in the AF2-predicted model were remarkably similar to the ones in the experimental structure (PDB: 1CIY) (Figure 1A). Similarly, AF2-predicted Cry4Ba showed very similar *α*-helical stretches to the crystal structure (PDB: 1W99), except at helix α3, which was extended N-terminally in the crystal structure. We note that in the original publication of Boonserm et al. [14], this ‘longer than expected’ *α*3 helix was attributed to the absence of N-terminal regions α1 and α2a resulting from enzymatic cleavage, which also produced a disordered α2b region (only residues 84–260 could be assigned). The accuracy of this interpretation is suggested by the fact that AF2 predicts a shorter α3 helix, more similar to that of Cry1Aa; thus, the absence of helices α1 and α2 leads to an N-terminal elongation of helix α3 towards *α*2b in the crystal structure. 

**Figure 2:**
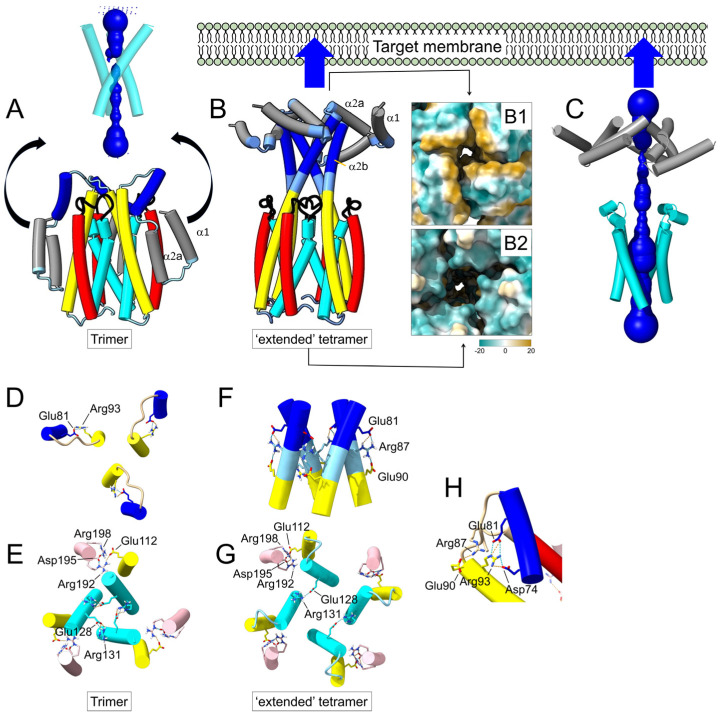
**AF2−predicted Cry1Aa conformational change in a transition trimer-to-tetramer:** (**A**) AF2-predicted trimeric structure of Cry1Aa domain I, and proposed movement of helices α1 and α2 away from the bundle. Helices α6 and α7 have been removed for clarity. HOLE profile is shown above; (**B**) AF2−predicted tetrameric ‘extended’ structure, where α3 forms a continuous α-helix with α2b and where α1 and α2a converge at the N-terminal tip of the oligomer that targets the host membrane. The central cavity is lined by helix α4 residues (cyan) away from the membrane and by helix α3 (yellow) closer to the membrane with residues Gln95 and Arg99. A view from the membrane-facing end shows that residues form a hydrophobic patch around the pore (gold), whereas, from the other end, residues from α4 are polar (cobalt blue). Plots corresponding to pLDDT and PAE for this tetrameric model are shown in Supplementary Figure S5; (**C**) HOLE profile of the model shown in (**B**); (**D**,**E**) top view of the main salt bridges in the trimeric model in (**A**) (helical fragments not involved in salt bridges have been removed for clarity), with salt bridge Arg93(α3)−Glu81(α2b) (**D**), and critical α4 residues (Arg131 and Glu128) involved in intermonomer contacts (**E**). Helix α3 has salt bridges via Glu112 with helix α6; (**F**,**G**) same as (**D**,**E**) for the tetrameric model in (**B**); a continuous helix α3-α2b is stabilized by salt bridges between Glu81, Arg87 and Glu90 (**F**), whereas Glu128 and Arg131 in α4 still stabilize weaker intermonomer contacts (**G**); (**H**) detail of salt bridges in the crystal structure of the Cry1Aa domain I monomer (PDB: 1CIY) in the α2b/α3 region.

Overall, the 3D structure of both toxins was almost indistinguishable from the crystal structures. For example, for domain I of Cry4Ba, α-carbon RMSD between predicted and crystal structure was 0.796 Å (146 pruned atom pairs out of 177 pairs) (Figure 1B) and 0.796 Å for Cry1Aa (184 pruned atom pairs out of 218 pairs) (Figure 1C). Even considering all three domains for Cry4Ba toxin, RMSD was only 0.8 Å (487 pruned atom pairs among 562 pairs) (Figure 1D,E). In addition, critical inter-monomeric salt bridges between Cry4Ba α4 helices were also present in the AF2-predicted model (Supplementary Figure S1). Cry1Aa also produced a trimeric model, shown in Figure 2A.

In summary, both Cry1Aa and Cry4Ba produced high-quality monomeric and trimeric models as judged by the two metrics provided by AF2: pLDDT and PAE. The pLDDT (predicted local distance difference test) is a *per-residue* confidence score (>90 = high confidence, and >50 = low confidence) [65,66]. Regions with pLDDT > 90 are expected to be modeled with high accuracy, whereas regions with pLDDT < 50 may represent an unstructured region or only structured as part of a complex. The predicted aligned error (PAE) (measured in Ångströms and capped at 31.75 Å) indicates the expected positional error at residue x if the predicted and actual structures are aligned on residue y. Thus, low PAE values (colored generally in blue in a PAE plot) between two domains or subunits represent well-defined relative positions and orientations of these two bodies. For both toxins, pLDDT and PAE plots are shown in Supplementary Figure S2.

### 2.2. Prediction of a Tetrameric Oligomer of Domain I in Cry1Aa Toxin

It has been proposed that the activated Cry toxins can form homotetramers in solution and that these tetramers may be responsible for the insertion of the toxin in membranes [55,68]. When we tested a tetrameric model of Cry1Aa domain I in AF2, the best model included an obvious reorganization of the N-terminal α-helices (Figure 2). Compared to the trimeric model (Figure 2A), helix α3 formed a continuous α-helix with helix α2b, whereas helices α1 and α2a clustered at the tip of a four-fold bundle (Figure 2B). It is worth noting that in most Cry toxins, α1 and α2a helices constitute the most hydrophobic part of the molecule, even more than helix α5 (Supplementary Figure S3), and in some cases, they are even predicted to form a transmembrane domain (Supplementary Figure S4). Therefore, it is plausible that a hydrophobic patch formed by these two helices (Figure 2B, panel B1) initially targets the host membrane. This dramatic conformational change could be triggered by the reorganization of domains I-III after the interaction of the toxin with a receptor. This ‘extended’ model consists of a central ~7 nm-long helical structure formed mainly by helices α4 to α2b. On the side farther from the membrane, the central lumen has a hydrophilic opening (Figure 2B, panel B2). This cavity is lined by α4 residues Glu128, Ile132 and Asp136 and polar residues Ser139 and Thr143, which also form the interface of the trimeric model (Figure 2A). Deeper into the interface, there is a positive charge (Arg99 from helix α3). However, helices α3 and α2b form a constriction incompatible with a channel (Figure 2C). In these trimeric and tetrameric models, intermonomer contacts are mediated mainly by helices α3 and α4. 

In the trimer (Figure 2A), apart from an intrachain salt bridge between α3 Arg93 and α2b Glu81 (Figure 2D), the predicted salt bridges between Arg131 and Glu128 of adjacent α4 helices suggest a strong interaction between the three monomers (Figure 2E). Another salt bridge is found between Glu112 (α3) and Arg192 (α6). 

In the tetrameric model (Figure 2B,C), the salt bridges between α3 and α6 are retained, but α3 and α2b form a continuous α-helix where Glu81 of one monomer interacts with Arg87-Glu90 of another (Figure 2F). Helices α4 appear to be more separated, and interaction between monomers is weaker than in the trimer (Figure 2G). It should be noted that in the crystal structure of Cry1Aa, obtained in monomeric form (PDB: 1CIY), domain I also contained intramolecular salt bridges between Glu81 and Arg93, connecting α3 and α2b (Figure 2H). 

We note that in this ‘extended’ tetrameric model, the distance between the α1/α2a helices and the α4/α5 hairpin is only ~3 nm. Therefore, this hairpin may eventually reach the membrane and insert, at which point the N-terminal helices, from α1 to α3, may rest on the membrane surface, whereas the rest of domain I inserts in the membrane. 

**Figure 3:**
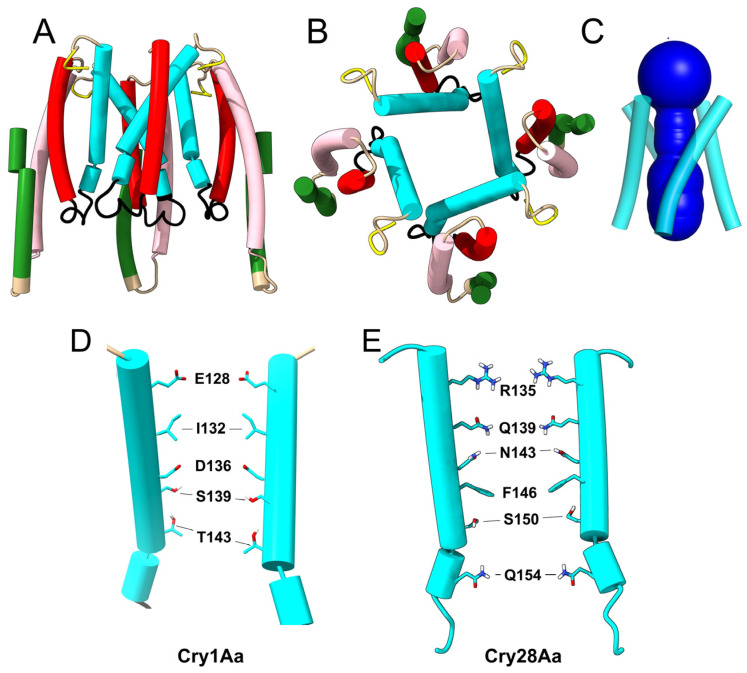
**Tetrameric ‘membrane-inserted’ model of Cry1Aa domain I:** (**A**–**C**) AF2-predicted tetrameric structure of domain I: side (**A**), top (**B**) and HOLE profile view (**C**); (**A**) helices 6 and 7 of one monomer were deleted for clarity; (**D**,**E**) helix α4 residues lining the channel in Cry1Aa (**D**) and Cry28Aa (**E**) in two opposed helices. Two monomers have been removed for clarity.

Accordingly, we also tested a tetrameric model of Cry1Aa after removing these N-terminal α-helices. In this model (Figure 3A–C), the central cavity was wider, with a minimum radius of 4.8 Å, and lined by the same α4 residues described for the ‘extended’ model (Figure 3D). According to this, we propose that the ‘extended’ tetrameric form (Figure 2B) is an intermediate preceding the membrane insertion of domain I helices α4 to α7. 

When a tetrameric model was tested for Cry4Ba, no ‘extended’ intermediate was observed, even after using a variety of sequence lengths and regardless of using ColabFold or a local installation of AF2. Structures were either not symmetric, clearly showing a preference for a trimeric organization, or showed interactions via helix α7, leaving α4 at the edges of the oligomer, large pores formed by domain III, dimer up–dimer down orientations and other nonsensible structures. Equally, no sensible tetrameric structure was obtained for Cry4Ba after removing the N-terminal α-helices. Attempts to obtain a tetrameric model with other toxins, e.g., Cry2Ab, Cry3Aa, Cry1Ia, or Cry11D, also failed. These toxins, including Cry4Ba, showed a clear preference for a trimeric organization, as reported before [52]. However, for Cry28Aa, although an extended model was not obtained, AF2 produced a tetrameric model after removing helices α1 to α3, similar to the one obtained for Cry1Aa (Figure 3E). 

For Cry1Aa, this channel shows an overall negative charge that is asymmetric (Figure 3D), negative farther from the membrane and positive at the end closest to the membrane. Although it is not possible to determine the selectivity of such a channel without considering all possible ionizable groups, including those not directly exposed to the channel lumen [69,70,71], the presence of two clear negatively charged rings lining the channel (formed by Glu128 and Asp 136) suggests that this side would attract extracellular cations into the channel, which together with the negative intracellular potential may produce an inwards cation flow. However, for Cry28Aa, the channel seems to have a positive charge at the lumen; therefore, ion selectivity may be species-dependent.

### 2.3. Prediction of Oligomers Formed by the α4–α5 Hairpin

As discussed above, it is conceivable that the binding of helices α1 and α2a in the target membrane ultimately facilitates the insertion of a loop formed by the α4–α5 hairpin (black loop in Figure 2B). Insertion of these helices would be in accordance with the ‘umbrella model’. However, insertion of just these two helices is in contrast with results where toxicity was not affected by, for example, α5–α7 crosslinking [39]. The latter suggests concomitant insertion of helices α4 to α7, but helices α6 and α7 are unlikely to remain buried in the membrane because they are not hydrophobic, leaving only the α4/α5 hairpin membrane inserted. 

Therefore, we tested whether AF2 could produce plausible α4–α5 hairpin oligomers that would be compatible with channel or pore formation. We tested Cry1Aa or Cry4Ba oligomers from *n* = 3 to *n* = 18, and the quality of the models was determined from the respective PAE plots, specifically low by off-diagonal PAE values (Supplementary Figure S6). The results for both sequences were similar, and only the results for Cry1Aa are described. A trimer of hairpins was clearly not a reliable model, but larger oligomers produced better PAE plots (*n* = 6) and peaked again for *n* = 14 and upwards (until *n* = 17). Quality decreased for *n* = 18, and no more oligomers were tested. In the small oligomers (*n* = 4 to *n* = 7), only helix α5 lined the lumen of the channel, which is incompatible with mutagenesis data [32,33,34,35,36]. Thus, these models may represent AF2-artefacts caused by the absence of helices α7 and α6. Supplementary Figure S7 shows a 6-mer as a representative of this group. 

**Figure 4:**
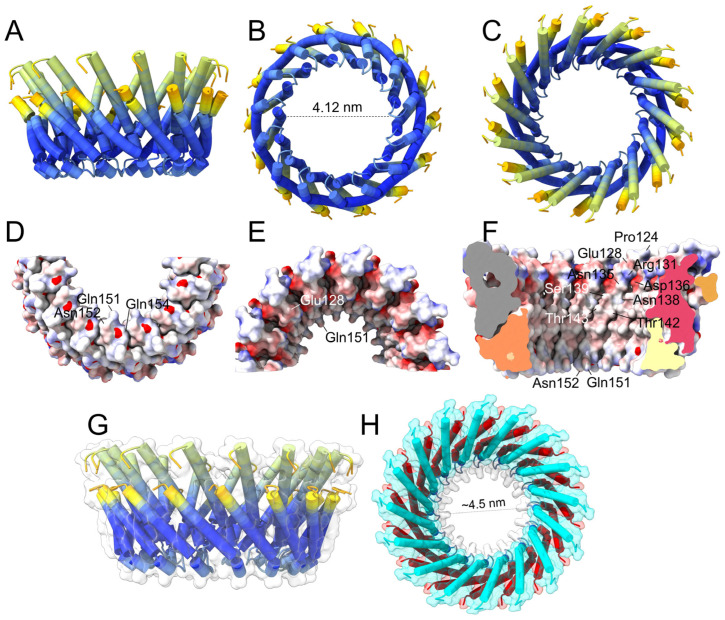
**Structure of Cry1Aa AF2-predicted larger oligomer:** (**A**–**C**) Predicted pore formed by 14 α4–α5 hairpins colored according to pLDDT in side (**A**), bottom (**B**) and top (**C**) views; (**D**–**F**) same as (**A**–**C**) for the 14-mer depicted as surface electric charge. The helix lining the pore is α4, and polar residues facing the pore are indicated. (**F**) Half of the pore was deleted for clarity. The pLDDT and PAE plots for the 14-mer are shown in Supplementary Figure S8; (**G**,**H**) predicted side view of pore formed by 16 α4–α5 hairpins colored according to pLDDT (**G**); top view (**H**) where helices α4 and α5 are labeled cyan and red, respectively.

In contrast, for the larger oligomers (*n* = 14 to *n* = 17), α4 was luminally located, and α5 faced the lipids (Figure 4). We chose the 14-mer as a representative of this group, which is shown colored according to the pLDDT (blue is the higher confidence; red is the lowest) (Figure 4A–C). Most of the hairpin is colored in deep blue, particularly the loop between α4 and α5, which supports the plausibility of the proposed model. The same model in three different orientations (Figure 4D–F) is represented (electric charge) where the position of luminal polar and charged residues is indicated, essentially the same as those described in Figure 3D. Increasing the number of monomers merely made the pore larger; for the 16-mer (Figure 4G,H), the diameter of the pore was about 4.5 nm, which may be able to allow the membrane passage of the three-domain activated toxin (approximate diameter of 5 nm). 

## 3. Discussion

### 3.1. AF2 Can Accurately Predict the Crystal Structures of Cry1Aa and Cry4Ba

Cry toxins are agriculturally and medically significant, as these toxins can protect crops from insect pests and control mosquito vectors of human diseases. Although several high-resolution structures of Cry toxins are available, the steps leading to membrane insertion and subsequent events are still unclear. Herein, we used AF2 to shed light on these steps. AF2 is still limited by the availability of diverse and sufficiently large MSAs and in its prediction of hetero-oligomers (e.g., toxin to its receptor) and effects of point mutagenesis, although recent advances are significant in these areas [72,73]. However, with good MSAs available, AF2 predicts monomeric and oligomeric protein models with striking accuracy, even for membrane proteins, as shown recently [74,75]. Also, AF2 can potentially produce models of conformational intermediates that can be validated experimentally using mutagenesis or structural techniques. 

**Figure 5:**
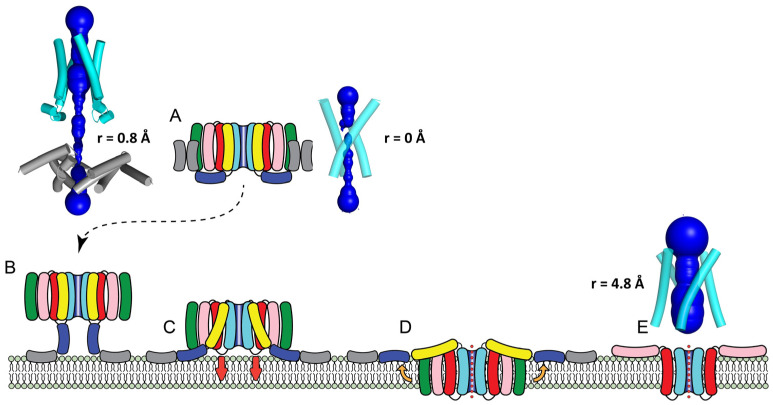
**Proposed mechanism of insertion of Cry toxins:** (**A**–**C**) The toxin is in a stable trimeric state in solution (**A**), and it may form tetramers after binding a receptor or the membrane. An unknown trigger straightens helix α3 towards α2b, forming an ‘extended’ model where hydrophobic helices α1 and α2a bind the membrane (**B**); the helix between α3 and α2b reverses its straight conformation and bends again, and the hairpin between helices α4 and α5 inserts into the membrane together with α6 and α7 producing the ‘membrane-inserted’ tetramer (**D**). The latter two helices are less hydrophobic and ‘float’ towards the membrane surface (orange arrows); the α4–α5 hairpin forms a tetrameric channel (**E**), and it may be enlarged by the addition of further hairpins, allowing translocation of toxin monomers to the host cytoplasm. (**E**) Helices α3 to α1 were deleted for clarity. HOLE profiles (dark blue) and minimum radius (r) are shown for the trimeric form (**A**), tetrameric ‘extended’ model (**B**) and tetrameric ‘membrane-inserted’ model (**D**,**E**).

In the present work, we first used AF2 to reproduce the structures of two representative Cry toxins, Cry1Aa and Cry4Ba, both as monomers or as homotrimers, using just domain I or domains I-III. These two toxins were solved by crystallography as a monomer (Cry1Aa) and as a trimer (Cry4Ba), but it has been reported that both may form trimers or tetramers after interaction with membranes or receptors. Consistent with this, trimers of both Cry1Aa and Cry4Ba were predicted with high confidence, where helices α3 and α4 of domain I are at the interface of the trimer (Figure 5A). 

### 3.2. The Extended Cry1Aa Tetrameric Intermediate

Since several lines of evidence also point to the possible formation of homo-tetramers representing pre-pore structures that insert into membranes, AF2 was used to predict a tetrameric form of these toxins. For Cry1Aa, but not for other toxins tested, a tetrameric model—although predicted with less confidence than the trimer—showed helix α3 extending N-terminally, forming a continuous α-helix with α2b, whereas helices α1 and α2a clustered at the tip of the tetramer, forming a hydrophobic patch (Figure 5B). In this model, helices α3 and α4 of domain I are at the interface of the oligomer, although this structure forms a central lumen that is too narrow to explain ion channel activity. The contribution of helix α4 [32,33,56,76] and helix α3 [35,42,77] to toxicity and oligomer formation is well supported experimentally [63,78,79]. For example, in Cry1A toxin, α3 mutation R99E inhibited oligomerization [80], and Arg99 is found at the interface of this proposed extended tetrameric model. However, neither Cry4Ba nor several other toxins (see Results section) produced such conformation. This AF2-predicted conformational change consisting of straightening of the region α2b–α3 is supported by a number of experimental observations. 

First, an N-terminally extended α-3 helix was observed in two trimeric Cry toxin crystal structures, Cry4Ba [14,15] and Cry5Ba [21], where N-terminal helices α1 and α2a were lost during crystallization, pointing to an inherent tendency of the sequence between α2b and α3 to become α-helical. 

Second, mutagenesis and biochemical data have strongly suggested that the link between helices α2b and α3 undergoes a conformational change important for toxicity [43,44,48]. For example, in Cry1A, mobility restriction experiments using disulfide bonds linking (α2b)−(α3) or (α2a)−(α3), or linking the loops at the end of α3 and α4, i.e., residue 88 (loop α2b/α3) and residue 153 (loop α4/α5), severely affected oligomerization and toxicity against larvae. These results suggested that helices α2b and α3 must undergo a conformational change via the formation of an extended α3 helix during the toxic activity of Cry1Ab and Cry1Aa [31,45]. This conformational change could be triggered by proteolysis and/or as a result of an interaction with a receptor, which could displace helix α1. Indeed, simple deletion of Cry1A helix α1 increased toxicity and oligomerization to resistant insects [35,42,77] and induced the formation of oligomeric structures even in the absence of a receptor [81]. 

Third, the loop between α2 and α3 has been suggested to interact with phospholipids in the membrane, as shown by inactive mutants A92D, A92E and R93F and the loop between α4 and α5 (Y153D) [53,54], which reduced irreversible binding, possibly reflecting a disturbed membrane insertion capability [53]. 

Fourth, in Cry3Aa, only the region encompassing helices α1–α3 experienced conformational changes after interaction with liposomes of synthetic lipids, and deletion of these helices led to orders-of-magnitude-reduced insecticidal activity [41]. FRET and electrical current measurements indicated that pore-forming activity required separation between helix α1 and α2a (residues 39 and 50) [42]. Mutagenesis data also support the involvement of helix α3 and α2b in the mechanism of pore or pre-pore formation; in Cry1A and Cry11, several mutants in α3 [81,82,83] and α2b (Pro70) [78,84] affected toxicity and oligomerization or reduced ion transport ability. 

Therefore, both experimental and prediction point to a conformational change in the N-terminal helices of domain I, but is this the final product, or just an intermediate to further conformational changes and insertion into the lipid bilayer? The first option has been suggested recently in the literature, where helices α1 to α4 would form a long extended helix and channel [27,43,45]. This idea is inspired by structural data from other toxins produced by some Bt strains. For example, cryo-electron microscopy of the vegetative insecticidal protein (Vip3) [5] was consistent with a conformational change where the N-terminal domain of Vip3Aa and Vip3Bc form a long four-helical coiled-coil α-helix needle required for membrane insertion [85,86]. In these toxins, it was proposed that the tip of this bundle, formed by helices α1 and α2, would insert into the membrane to form a pore, but these regions were not resolved in the structure. The very long distance (~20 nm) between the core of the protein and the membrane plane would be occupied by membrane receptors. Similarly, in the toxin complex (Tc), also present in Bt strains, an α-helical needle spans the membrane, and a flexible tip formed by a small α-helix is involved in membrane penetration and pore formation [87,88]. Clearly, the AF2 prediction extension of helix α3 and α2b reported here and the formation of a tetrameric bundle with helices α1 and α2a at its tip is reminiscent of such models. 

### 3.3. The Cry1Aa Tetrameric Membrane-Inserted Channel

That the extended model described above is an intermediate before membrane insertion is suggested by experimental data consistent with toxicity being dependent on the separation of helices α3 and α4 [39,40,42,43,44,45]. Indeed, bonds between Cry1Aa helices α3 and α4 resulted in lower toxicity and pore-forming activity [39]. Second, in Cry1Aa, the separation between the α4/α5 loop and the N-terminal helices α1/α2a is only ~3 nm, which is a relatively short distance for this α4/α5 loop to negotiate before membrane insertion, especially considering the flexibility between α2b and α3 helices found in the trimeric structure. Third, an extended channel as a final structure is inconsistent with the reported buried/inaccessible location of the four helices α4–α7. Enzymatic digestion experiments in Cry1Ac have shown that only α1 was fully exposed to the action of protease K when the toxin interacted with membranes [37]. In Cry1Aa, helices α2 to α7, and also domain III, were protected from enzymatic digestion [89]. Fluorescence quenching data show that domains II and III are partially exposed to the solvent [90]. In addition, fluorescently labeled residues in N-terminal helices showed close FRET contacts with fluorescently labeled lipid polar headgroups, but data for residues in helices α4 to α7 (and domain III) were more consistent with a farther distance from the lipid polar head groups and a membrane-inserted topology [45]. Finally, bonds between helix α5 with α6 (or with α7) or between helix α7 and domain II did not affect toxicity [39]. Overall, these data are consistent with a conformational change involving helices α3 and α4, presumably resulting in the separation of helices α1–α3 from the rest of the bundle in domain I. This separation could then trigger the coordinated insertion of helices α4–α7 into the membrane (Figure 5C,D). Accordingly, we tested whether AF2 could produce a tetrameric model of domain I in the absence of helices α1 to α3. 

The model obtained for Cry1Aa has residues Glu128, Ile132, Asp136, Ser139 and Thr142 lining the lumen of this channel, which is much wider (Figure 5E) than the one present in the extended model (Figure 5B). Mutations at these residues in Cry1A eliminated both toxicity and pore-forming activity [32,33,34,35,36,38,76]. In this tetrameric model, helix α5 is located away from the lumen of the channel; however, due to its proximity, some mutations in α5 can potentially affect the toxicity and channel stability. For example, Asn 183 in α5 of Cry4Ba was essential for oligomerization and toxicity [57] but not mutations at other α5 polar residues. His168 in Cry1Ab α5 has also been proposed to have luminal orientation on similar grounds [35,91]. It is known that Cry toxins produce multiple, non-specific conductance states, as shown when toxin monomers are incubated with receptor-free membranes. Cry toxins can also show large conductance states in Cry1Ac, Cry3A, Cry3B and Cry1C in synthetic planar lipid bilayers [92] with an estimated pore size of 1–2 nm [53,54] formed by 4 to 6 toxin monomers. The pore formed by the AF2-predicted tetramer has an average diameter of 2 nm and a smallest constriction of ~1 nm.

### 3.4. Pores Made of α4/α5 Hairpins

Once helices α4 to α7 are inserted in the membrane, it is possible, based on the umbrella model, that helices α6 and α7 eventually ‘float’ due to their hydrophilicity and lay parallel to the membrane surface. The α4–α5 hairpins could form a tetrameric oligomer or grow to form larger aggregates. The latter could be similar to the 14–17mer obtained in AF2, with helix α4 in a luminal orientation, reminiscent of the multimeric pore proposed for Cyt1Aa, involving an indeterminate but large number of subunits [93]. The formation of such a large pore may contribute to osmotic lysis, or it may be able to facilitate the entry of toxins into the cell. 

### 3.5. Testing the Validity of the Tetrameric Models for Cry1Aa

Given the large body of existing experimental data for Cry1A toxins, which is generally consistent with the tetrameric models suggested here, it is difficult to propose additional mutagenesis data that would ‘disprove’ these models. Mutations that would support such models are suggested by the stabilizing salt bridges shown in the Results section. However, given that these models are tetrameric, such oligomeric form should be observed in preparations of the toxin in the presence of mild detergent (not SDS) using analytical ultracentrifugation (AUC), X-ray diffraction (XRD) or cryo-electron microscopy (Cryo-EM). In the presence of lipids, XRD, Cryo-EM or electron paramagnetic resonance (EPR) could be used. However, a definitive answer can only be obtained in cryo-EM studies that involve the toxin, its receptor and lipids that mimic the native membranes. 

## 4. Materials and Methods

### Sequences of Cry Toxin and Structure Prediction

Structural models were obtained with ColabFold (AlphaFold2 using Mmseqs2) [61] that uses AlphaFold-2 [62]. The best structure was minimized with Amber (*use_amber* = True). Other parameters were *template_mode* = None, *msa_mode* = MMSeqs2 (Uniref + Environmental), *pair_mode* = unpaired + paired, *model_type* = auto, *num_recycles* = 6 and number of seeds = 3. For each prediction, the best models (rank 1) were selected according to average pLDDT, and complexes were sorted by pTMscore, which reports on the accuracy of prediction within each protein chain. Predictions were performed mainly using the toxin sequences of Cry1Aa (>sp|P0A366|) or Cry1Ab (>sp|P0A370), with only a six-mutations difference in domain I, and Cry4Ba (>sp|P05519|), either using domains I–III or domain I alone. The N-terminal end (~35 residues) was found to be very flexible and had a very low pLDDT score; therefore, it was deleted without change in the predicted models. The validity of the models was determined by their low PAE score. Oligomeric forms of these two toxins were also tested using a local installation of AF2 (commit 7c9114c, 10 August 2023) [62]. In this local installation, predictions were made using the multimer mode [94]. The default version of the full dataset, 5 seeds and the top-ranked structure were selected. For the predictions consisting of the hairpin formed by α4 and α5 helices, we used the corresponding sequences of Cry1Aa and Cry4Ba. For Cry1Aa, PTNPALREEMRIQFNDMNSALTTAIPLFAVQNYQVPLLSVYVQAANLHLSVLRDVSVFGQ. Oligomers of this hairpin were tested from *n* = 3 to *n* = 18. Analysis of structures and helix segment quantification was performed using SWISS-MODEL (https://swissmodel.expasy.org), accessed on 1 September 2023. Monomeric forms were refined using the online tool ModRefiner (https://zhanggroup.org/ModRefiner/) [95], whereas multimers were refined using Amber in ColabFold, as described above. Graphical representation, molecule visualization and search of salt bridges were performed in Chimera X [67,96]. Channel diameter profiles are based on the Van der Waals radius generated using the HOLE software v2.2.005 [97] on the lowest-energy structures. The HOLE spherical probe radius was set to 5–10 Å, and models were visualized in PyMOL v2.3.4.

## 5. Conclusions

- AF2 predicts the structures of three-domain Cry toxins with high accuracy.
- For Cry1Aa, AF2 predicted two tetrameric forms: (1) an ‘extended’ intermediate and (2) a membrane-inserted model, which would be responsible for channel activity.
- We provide a detailed description of the residues in α4 that line the lumen of the Cry1Aa tetrameric channel, which are in agreement with experimental data.
- AF2 predicts an intriguing large ensemble of α4–α5 hairpins that may be part of the toxicity mechanism. This large pore may contribute to osmotic lysis, or it may be able to facilitate the entry of toxins into the cell, leading to disruption of homeostasis and death.

## Data Availability

Data are contained within the article.

## Supplementary Materials

The following supporting information can be downloaded at: https://www.mdpi.com/article/10.3390/ijms242316809/s1.
