# Supplementary material for: Channel Formation in Cry Toxins: An Alphafold-2 Perspective
Source: Int J Mol Sci. 2023 Nov 27;24(23):16809. doi: 10.3390/ijms242316809 (PMC10705909; doi:10.3390/ijms242316809)
Supplement: Supplementary file 1 [file ijms-24-16809-s001.zip › ijms-2705054-supplementary.pdf]

Supplementary File.

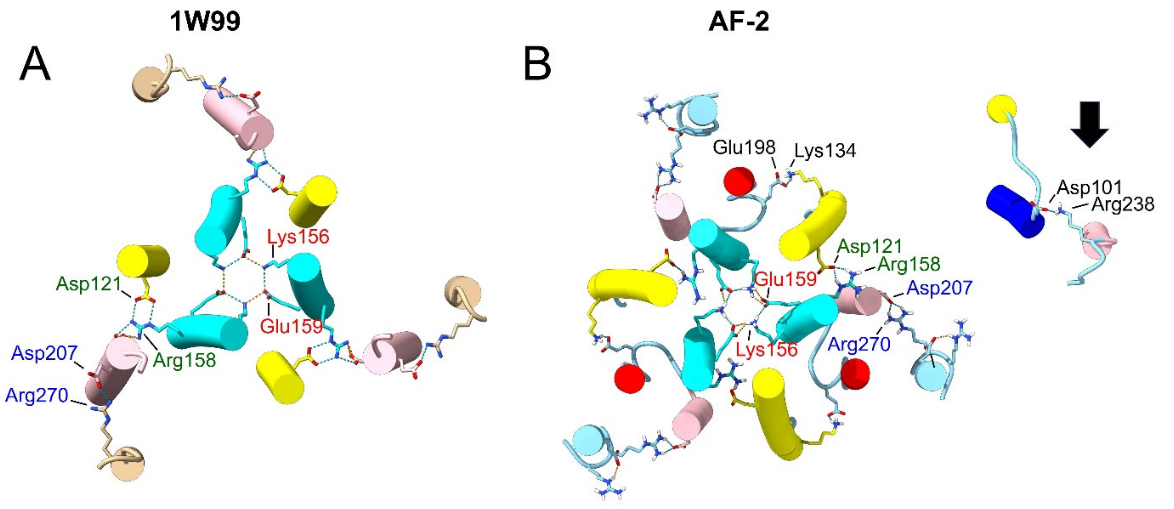

Supplementary Figure S1. Comparison of critical salt bridges in domain I between crystal and AF2-predicted Cry4Ba trimeric structures. (A) Top view of Cry4Ba domain I trimer (PDB:1W99) showing only the parts of the molecule involved in salt bridges; (B) same as (A) for the AF2-predicted structure. Three salt bridges common to the two structures are shown in the same label color (red, blue and green) and only one is inter-monomeric (red), involving α4 residues Lys156 and Glu159. In both models, helix α4 also has salt bridges via Arg158 with helices α3 and α6. Arrow: in the N-terminal fragment that was missing in the 1W99 crystal structure, AF2 finds another salt bridge, between Asp101 (α2b) and Arg238 (α6).

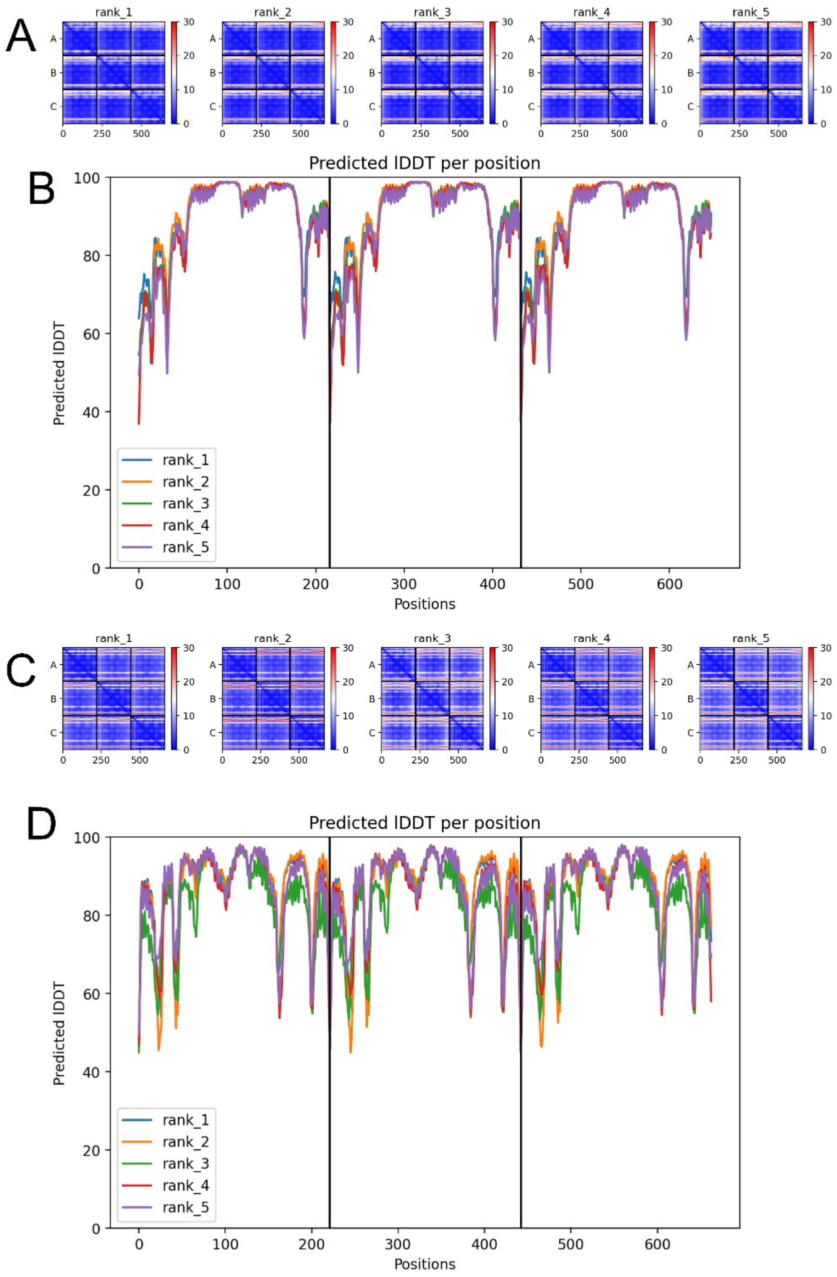

Supplementary Figure S2. Quality of trimeric domain I models of Cry1Aa (residues 35-250) and Cry4Ba. (A) PAE plots for models rank 1-5; (B) same as (A) showing a pLDDT plot, which is lower for the first ~36 residues (equivalent to residues 51-87, i.e., α2a and α2b); (C-D), same as (A-B) for Cry4Ba (40-250).

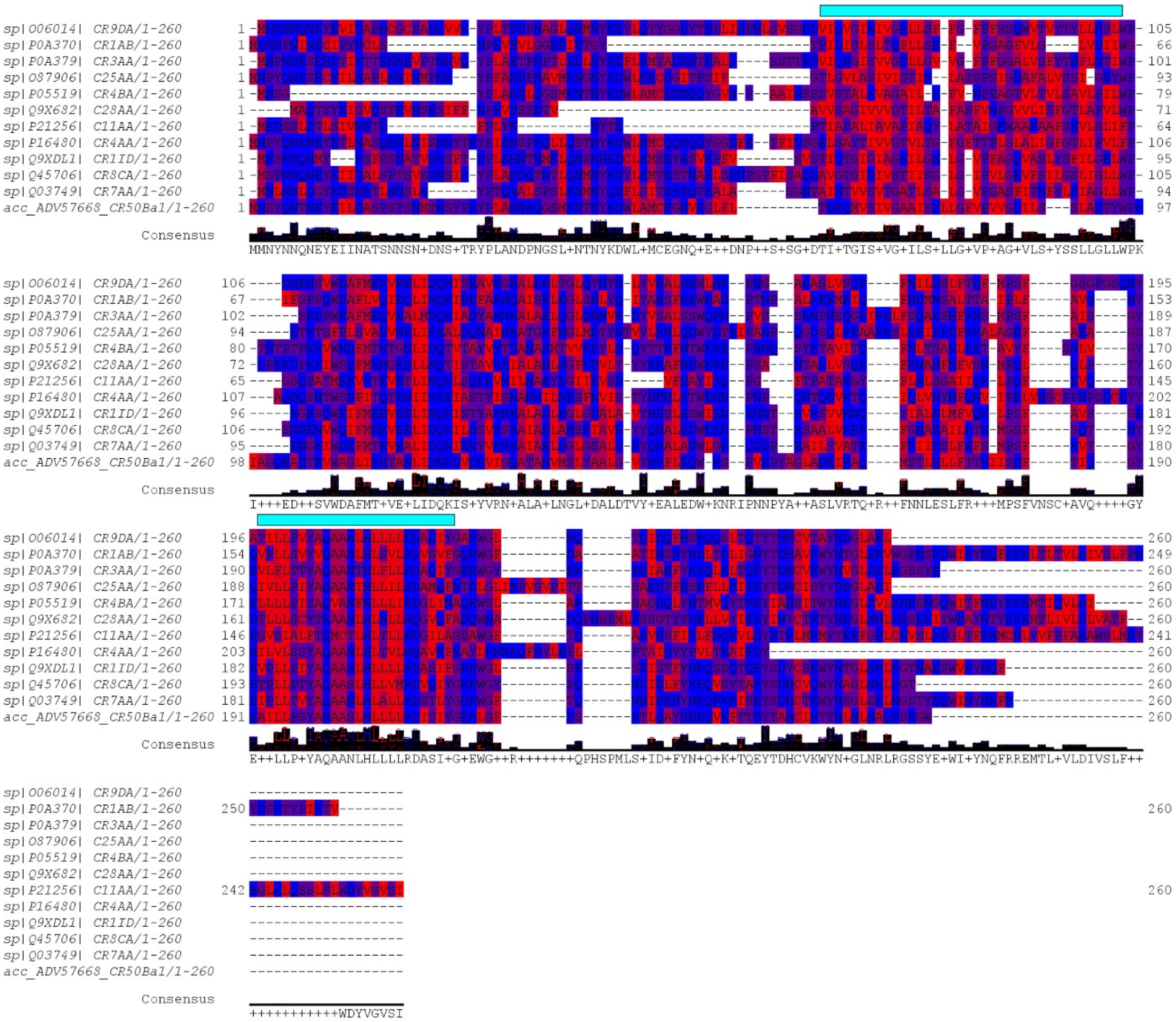

Supplementary Figure S3. Hydrophobicity output generated in Jalview for selected Cry toxins (Swiss Prot code indicated, except for Cry50Ba1 which only has GenBank accession number). Red: hydrophobic; blue, polar. The two most hydrophobic regions (α1-α2 and α5) in all sequences are below a cyan bar.

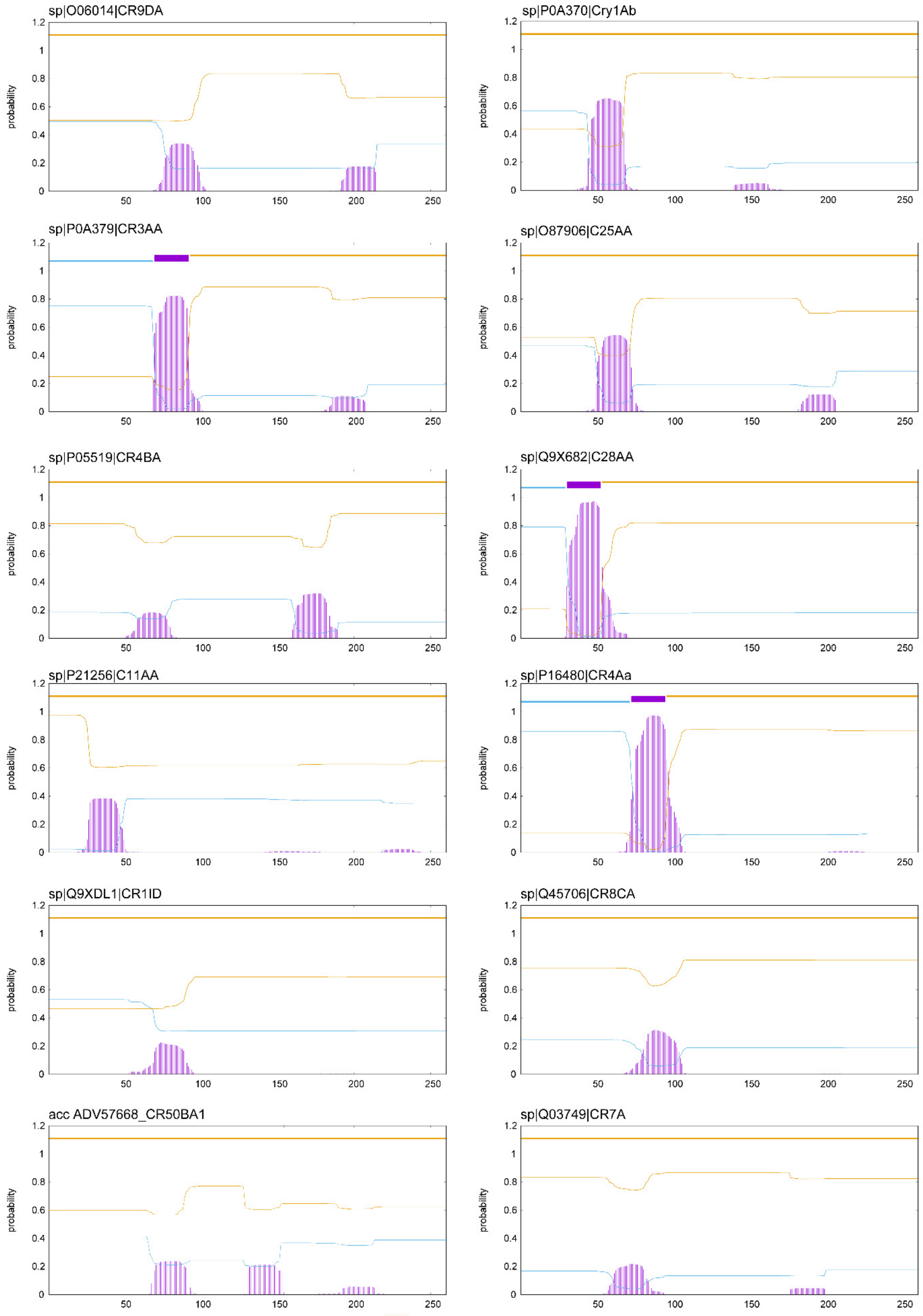
sp|O06014|CR9DA
sp|P0A370|Cry1Ab
sp|P0A379|CR3AA
sp|Q87906|C25AA
sp|P05519|CR4BA
sp|Q9X682|C28AA
sp|P21256|C11AA
sp|P16480|CR4Aa
sp|Q9XDL1|CR1ID
sp|Q45706|CR8CA
acc ADV57668_CR50BA1
sp|Q03749|CR7A
probability

Supplementary Figure S4. TM domain prediction (purple) by TMHMM for Cry toxins shown in Supplementary Fig. S3 (Swiss-Prot code indicated). The codes were chosen from the list 'List of Bacillus thuringiensis Holotype Toxins', except for Cry50Ba1 (Cry50-like protein) which only has GenBank accession number. Blue and yellow lines represent the probability of cytoplasmic or lumenal (extracelular) topology, respectively.

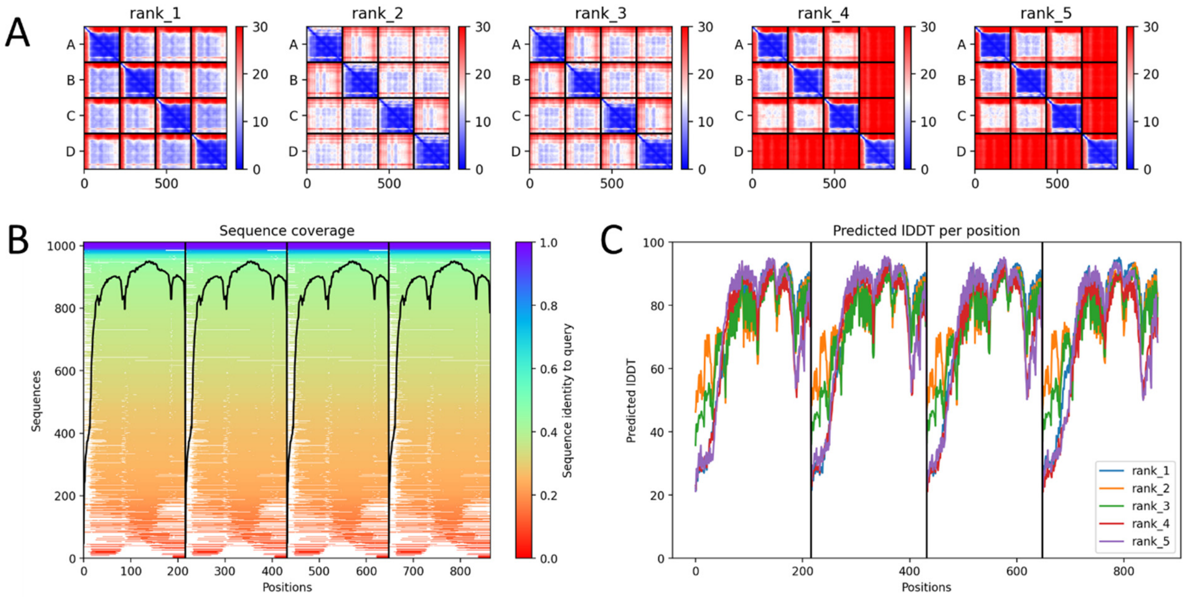

Supplementary Figure S5. AF-2 structure prediction of the tetrameric Cry1Aa domain I (35-250). (A) PAE plots for the best 5 models (rank 1-5). The PAE scale is shown on the right where low (blue) is optimal and high (red) is unsuitable; (B) MSA; (C) pLDDT.

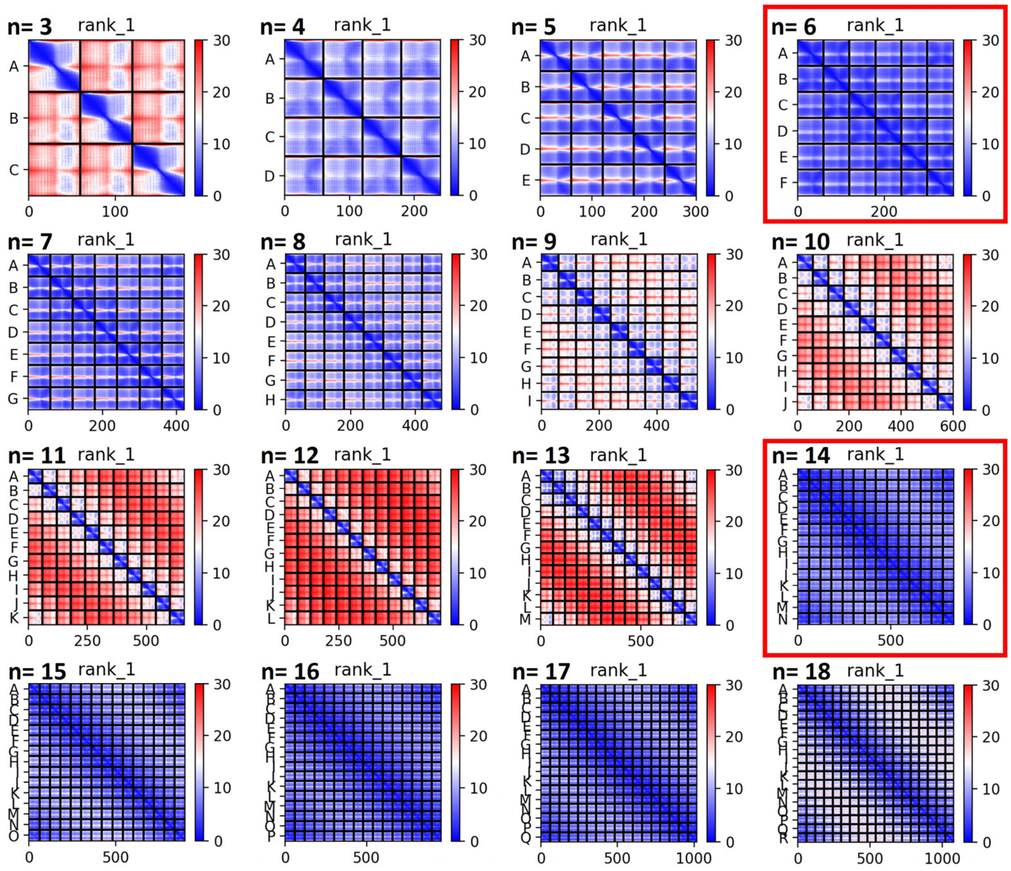

Supplementary Figure S6. AF-2 structure prediction of oligomers of Cry1Aa helix 4 and 5 hairpin. PAE plots for the best (rank 1) models of sizes from 3 to 18. The Predicted Alignment error (PAE) scale is shown on the right where low (blue) is optimal and high (red) is unsuitable. Optimal plots (n = 6, 14) are highlighted.

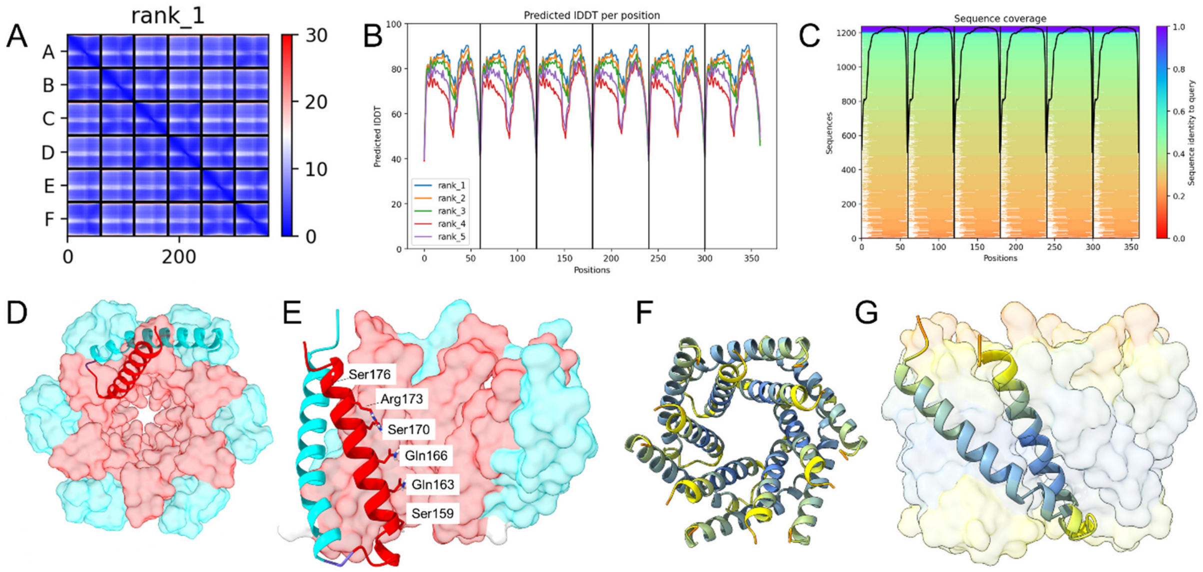

Supplementary Figure S7. AF-2 structure prediction of hexameric Cry1Aa α4-α5 hairpin. (A) PAE plots for the best model. The PAE scale is shown on the right where low (blue) is optimal and high (red) is unsuitable; (B) pLDDT; (C) MSA; (D) hexameric pore formed by α4 (cyan) and α5 (red) α-helices of Cry1Aa, where the latter is lining the pore; (E) side view, with polar residues of α5 lining the pore as indicated, and where three monomers have been removed for clarity; (F) helix tilt of helices α4 and α5 in the hexameric model.

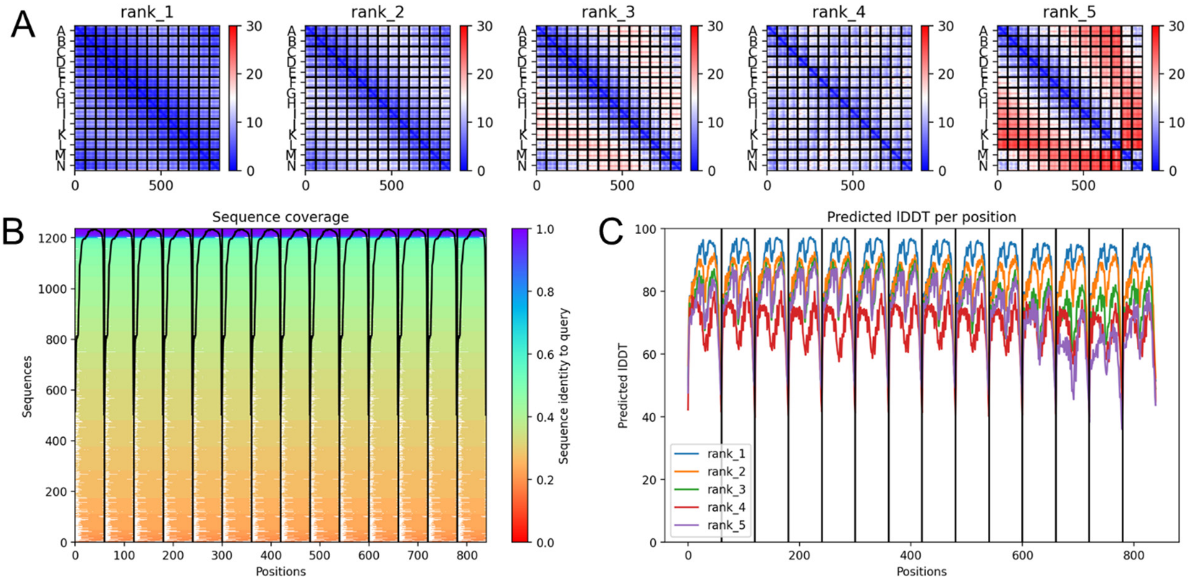

Supplementary Figure S8. AF-2 structure prediction of Cry1Aa 14-mer α4-α5 hairpin. (A) PAE plots for the best model. The PAE scale is shown on the right where low (blue) is optimal and high (red) is unsuitable; (B) MSA; (C) pLDDT.
